# From macro- to microfactors in health: Social science approaches in research on sexually transmitted infections

**DOI:** 10.1371/journal.pmed.1002490

**Published:** 2018-01-10

**Authors:** Ruth Kutalek, Elena Jirovsky, Igor Grabovac

**Affiliations:** 1 Unit Medical Anthropology and Global Health, Department of Social and Preventive Medicine, Medical University of Vienna, Vienna, Austria; 2 Department of General Practice and Family Medicine, Medical University of Vienna, Vienna, Austria; 3 Unit Lifestyle and Prevention, Department of Social and Preventive Medicine, Medical University of Vienna, Vienna, Austria

## Abstract

Ruth Kutalek and colleagues share their Perspective on Kipruto Chesang and colleagues' qualitative study of beliefs and practices among healthcare providers managing STIs in Kenya and discuss the value of this type of research for addressing biosocial challenges.

Perceptions of the body and sexuality are socially and culturally constructed. They are deeply influenced by historical realities, ethnic affiliation, power relations, gender roles, and concepts of morality. In the highly sensitive context of sexually transmitted infections (STIs), social science research that employs qualitative approaches is therefore especially important. It provides critical perspectives on multiple levels and takes into account biological, social, and political issues of STI coinfection or “syndemics” [1]. It also uses participatory approaches that empower patients and communities and enable culturally sensitive responses to infectious disease threats.

In a Research Article published last month in *PLOS Medicine’s* Collection on prevention, diagnosis, and treatment of STIs, Kipruto Chesang and colleagues [2] report on their qualitative study of healthcare providers (HCPs) working in HIV clinics in Kenya. Their study investigates HCP knowledge of STIs, day-to-day STI-related screening and treatment practices, HCP attitudes about STIs, health-seeking behavior of people living with HIV (PLWHIV), and structural factors affecting the management of STIs in this setting. The results are anticipated to provide input to Kenya’s updated national STI guidelines in 2018.

The authors conducted 87 in-depth interviews with HCPs in different professional roles, including clinical staff, pharmacy staff, and facility managers. The majority of the responses suggested that screening for STIs was done inconsistently or not at all. Reasons given included high workload, HCPs’ discomfort in discussing sexual issues, and HCPs’ anticipation of patient nondisclosure due to stigma. HCPs reported a widely held belief that STIs are a punishment for immoral behavior and felt that, fearing stigma, patients were less likely to disclose symptoms. HCPs sometimes blamed patients for acquiring STIs as this meant patients had not adhered to HCPs’ advice regarding safe sex practices. Supportive supervision and training on STIs was almost nonexistent, which resulted in a lack of confidence to discuss broader issues of sexuality and STIs with patients. HCPs felt that because of the strictly vertical character of HIV programs, STI and HIV services were not integrated, leading to poor clinical management of STIs. Other barriers to STI care included lack of accessibility and availability of drugs, costly treatment, and worries regarding privacy and patient confidentiality.

The article by Chesang and colleagues reveals, from the perspective of HCPs, the multilayered challenges and barriers to STI care that occur in a resource-poor setting. They report on problems on different structural levels, including infrastructure, human resources, and integration of system, that need to be considered holistically. The highlighted problems require a multidisciplinary analysis that combines social sciences—anthropology, political sciences, economics, and social history—with fields like epidemiology and clinical practice, an analysis “that permits us to take a properly biosocial approach to what are, without exception, biosocial problems” [3].

Chesang and colleagues provide an apt example of problems arising from vertical health programming. On the one hand, vertical programming provides much-needed financial resources where direly needed, as for HIV and tuberculosis (TB). On the other hand, this structure is often not flexible enough to target issues outside the program’s remit, thus creating parallel structures and often diverting human and economic resources away from horizontal healthcare services. Researchers are concerned about these resource flows and their implications for global health [4]. The findings of Chesang et al. clearly show how vertical programming can create inequity by disease in STI care, favoring HIV over other STIs. With HIV being an STI, the solution would be to integrate STI services in primary care and to provide free STI treatment.

The interviews also touched on interpersonal and cultural issues, as can be expected in the sensitive area of STIs. Interestingly, the study did not yield some of the themes uncovered in prior work, such as the importance of clear patient–HCP communication to facilitate sound, inclusive decision making [5] or the value of developing a trustful relationship that fosters confidentiality and respect [6]. The findings also did not provide detail on how communication with patients is taught in medical education.

Questions of morality and being a “good patient” are almost always pertinent in STI research [7–8]. Likewise important are questions of sexuality and gender relations (e.g., homosexuality), of stigmatization in communities and in healthcare facilities (e.g., blaming of patients), and of partner disclosure [9] and how all of these aspects influence the perception of STIs. All these topics emerge in this thematic analysis; interestingly, however, the solutions suggested by HCPs seem centered on structure and resources rather than HCP and community attitudes about STIs. This focus, in itself, warrants a deeper analysis of the meanings of sexuality in this setting.

More social science research on community perspectives, particularly on risk perception and prevention of STIs, is urgently needed, as STI treatment and care needs to be adapted to needs pertaining to rising incidences of resistances both to antiretroviral therapy (ART) and antibiotics. Increasing the role and visibility of in-depth studies would allow analysis of the intricate entanglement of challenges on structural and communicative levels and would help to adjust interventions to individual and community needs [10].

## Abbreviations

ART: antiretroviral therapy
HCP: healthcare provider
PLWHIV: people living with HIV
STI: sexually transmitted infection
TB: tuberculosis
